# Angiopoietin-Like-4, a Potential Target of Tacrolimus, Predicts Earlier Podocyte Injury in Minimal Change Disease

**DOI:** 10.1371/journal.pone.0137049

**Published:** 2015-09-09

**Authors:** Jian-Si Li, Xiao Chen, Lei Peng, Shi-Yao Wei, Shi-Lei Zhao, Tian-Tian Diao, Yi-Xin He, Fang Liu, Qiu-Ju Wei, Qing-Fang Zhang, Bing Li

**Affiliations:** 1 Department of Nephrology, Second Affiliated Hospital of Harbin Medical University, Harbin, People’s Republic of China; 2 Sichuan Academy of Medical Sciences & Sichuan Provincial People’s Hospital, Chengdu, People’s Republic of China; University of Houston, UNITED STATES

## Abstract

Podocyte injury plays central roles in proteinuria and kidney dysfunction, therefore, identifying specific biomarker to evaluate earlier podocyte injury is highly desirable. Podocyte-secreted angiopoietin-like-4 (Angptl4) mediates proteinuria in different types of podocytopathy. In the present study, we established an experimental minimal change disease (MCD) rat model, induced by adriamycin (ADR) and resulted in definite podocyte injury, to identify the dynamic changes in Angptl4 expression. We also investigated the direct effects of tacrolimus on Angptl4 and podocyte repair. We determined that the glomerular Angptl4 expression was rapidly upregulated and reached a peak earlier than desmin, an injured podocyte marker, in the ADR rats. Furthermore, this upregulation occurred prior to heavy proteinuria and was accompanied by increased urinary Angptl4. We observed that the Angptl4 upregulation occurred only when podocyte was mainly damaged since we didn’t observe little Angptl4 upregulation in MsPGN patients. In addition, we observed the glomerular Angptl4 mainly located in injured podocytes rather than normal podocytes. Moreover, we found that tacrolimus treatment significantly promoted podocyte repair and reduced glomerular and urinary Angptl4 expression at an earlier stage with a significant serum Angptl4 upregulation. And similar results were confirmed in MCD patients. In conclusion, this study represents the first investigation to demonstrate that Angptl4 can predict podocyte injury at earlier stages in MCD and the identification of earlier podocyte injury biomarkers could facilitate the prompt diagnosis and treatment of patients with podocytopathy, as well as determination of the prognosis and treatment efficacy in these diseases.

## Introduction

Proteinuria is a common clinical manifestation of kidney damage including glomeruli, tubules and microvessels et al. Albuminuria can be occurred when glomerular charge/size barrier injury including podocytes, endothelial cells and the glomerular basement membrane. Proteinuria is typically a reflection of increased glomerular permeability for albumin and other plasma macromolecules, and it is one of the most significant symptoms in kidney disease and is closely related to podocyte injury[1].Therefore, both proteinuria and albuminuria are nonspecific biomarker for podocyte injury. Minimal change disease (MCD), which is a type of podocytopathy, accounts for 10–15% of nephrotic syndrome cases in adults and is the leading cause of childhood nephrotic syndrome[2]. The typical clinical manifestation of MCD consists of substantial amounts of proteinuria, and it is always accompanied by serious podocyte foot process effacement. In general, corticosteroid therapy is effective for MCD patients. However, this therapy is ineffective in some MCD patients, and it cannot be considered as a treatment for some patients due to its multiple side effects[3,4]. There is controversy about whether MCD progresses to focal segmental glomerular sclerosis (FSGS) or is a separate disease from FSGS. If podocytes cannot be adequately repaired, which results in podocyte number loss, MCD may progress to FSGS, one of the leading causes of end-stage renal disease. Podocyte injury occurs in many forms of human and experimental glomerular disease, and it also plays central roles in proteinuria and kidney dysfunction[5]. Therefore, identifying specific biomarker to evaluate earlier podocyte injury is highly desirable. The identification of earlier targets of podocyte damage or novel specific biomarkers of earlier podocyte injury could facilitate the prompt diagnosis and treatment of patients with podocytopathy as well as the determination of the prognosis and treatment efficacy in these diseases.

Angiopoietin-like-4 (Angptl4) is a potent inhibitor of lipoprotein lipase[6] and induces marked hypertriglyceridemia after intravenous injection or adenovirus-mediated expression[7]. Angptl4 is highly expressed in the liver and adipose tissue; however, it is expressed at lower levels in cardiomyocytes, skeletal muscle and the kidneys[8,9]. Recent research has demonstrated that glomerular Angptl4 secreted by podocytes is upregulated in experimental MCD and membranous nephropathy (MN) rats, and research has shown that Angptl4-transgenic rats exhibit high levels of proteinuria, thus indicating that glomerular Angptl4 mediates proteinuria in some types of glomerulonephropathy[10]. In our previous study, which investigated a passive Heymann rat model, we demonstrated that the calcineurin inhibitor tacrolimus reduced proteinuria accompanied by decreased Angptl4 expression[11]. Moreover, increased glomerular and urinary Angptl4 expression have been identified in diabetic rats in our recent studies[12]. MN not only involved podocyte injury, but also accompanied with subepithelial immune deposits and an expanding glomerular basement membrane. Therefore, we explored the specific podocyte injury model, adriamycin (ADR) induced rat model, to investigate the relationship between Angptl4 expression and podocyte injury in this study. Desmin is an intermediate filament that typically indicates podocyte injury in various of experimental rat models[13,14], and synaptopodin is an actin-associated cytoskeleton in differentiated podocytes, which is typically used in normal podocyte evaluation[15]. Here, we investigated if Angptl4 reacted earlier to podocyte damage than the two previously described markers and if it could provide a new clue for the evaluation of podocyte injury and prompt treatment during an earlier stage.

Tacrolimus is a macrolide lactone antibiotic that is mainly used as an immunosuppressant to treat patients who receive allogeneic organ transplants or have autoimmune diseases. Some investigations have demonstrated that the calcineurin inhibitor, cyclosporine A (CsA), may act on the podocyte actin cytoskeleton to reduce proteinuria and protect kidney function[16]. Calcineurin is a central signaling controller in eukaryotes[17]; thus, tacrolimus treatment often results in hypertension, pathoglycemia and other multi-systemic side effects[18,19]. In recent years, there have been several reports of successful treatment with tacrolimus in patients with refractory MCD[20,21]. Therefore, investigation of the downstream targets of tacrolimus in MCD may facilitate the development of novel therapeutic agents for refractory MCD in clinical therapy. Our previous study demonstrated that tacrolimus may act on Angptl4 in podocytes to reduce proteinuria in MN[11]; thus, we postulated that it may have therapeutic effects on proteinuria and renal damage by acting on Angptl4 in the glomeruli in an ADR model.

In the present study, we established ADR nephropathy, which is a typical animal model of MCD[22] in rats, to identify dynamic changes in Angptl4 and to investigate the direct effects of tacrolimus on Angptl4 and podocyte repair.

## Materials and Methods

### Animals and experimental design

The animal experiments were performed in strict accordance with the National Institutes of Health Guidelines for the Care and Use of Laboratory Animals. The Animal Experiments Committee of Harbin Medical University approved all animal care and experimental procedures.

Eight-week-old male Sprague-Dawley rats (Harbin Medical University Second Affiliated Hospital Laboratories) weighing 200–220 g were used in this study. All rats were housed in an air-conditioned room and were provided free access to food and water (22±2°C; 12:12-hour light:dark cycle). The rats were euthanized under anesthesia (10% chloral hydrate by peritoneal injection), and all efforts were undertaken to minimize pain and discomfort.

The controls comprised rats injected with normal saline (n = 10). Nephrotic syndrome was induced via the venous administration of ADR (7.5 mg/kg body weight; Solarbio, Beijing, P.R.C., n = 60)[23]. Nephrotic syndrome was confirmed 14 days later via urinary protein measurement. Twenty ADR nephrotic rats were continuously treated with tacrolimus (1 mg/kg/day via the stomach; Astellas, County Kerry, Ireland) when proteinuria was present until euthanasia. The rats were randomly divided into two time point groups as follows: day 21 and day 28 (n = 10 per group). The remaining ADR rats were administered an oral dose of normal saline (5 ml/kg/day via stomach) and randomly divided into four time point groups as follows: day 10, day 14, day 21 and day 28 (n = 10 per group).

### Sample collection and proteinuria and serum parameter measurements

Twenty-four-hour urine samples were collected from individual rats housed in metabolic cages with free access to water but without access to food. Proteinuria was determined using the nephelometry method (Siemens BN II, Deerfield, IL, USA). On days 10, 14, 21 and 28 after ADR induction, 10 rats per group were euthanized under anesthesia. Kidney tissue and blood samples were obtained from the anesthetized rats. The blood samples were immediately analyzed with an automatic biochemistry analyzer (Roche, Cobasc 311, Mannheim, Germany) to measure the serum levels of albumin, total protein, triglycerides and cholesterol[24]. The renal tissues were processed for morphological studies, immunofluorescence microscopy and molecular biology experiments.

We conducted our human subject research with the approval of the Institutional Review Board of the Second Affiliated Hospital of Harbin Medical University in Harbin, China. All participants provided written informed consent according to the latest version of the Helsinki Declaration on human research ethics.

Human kidney tissues were collected during renal biopsy at the Second Affiliated Hospital of Harbin Medical University and were processed for immunofluorescence staining as subsequently described. Human urine was collected for 24 hours prior to treatment.

### Morphological studies by using transmission electron microscopy

Blocks of renal cortex tissue (1 mm^3^) were fixed as previously described by our group [11]. The blocks were subsequently examined and photographed using a Hitachi 7650 transmission electron microscope (Tokyo, Japan).

### Apoptosis assay

Renal tissues for light microscopy were fixed in 10% neutral buffered formalin for 24 hours, dehydrated, embedded in paraffin, and sectioned at 2 or 4 μm for terminal deoxynucleotidyl transferase-mediated dUTP nick end labeling (TUNEL). TUNEL was performed using an *in situ* Cell Death Detection Kit (Roche, Indianapolis, IN, USA) according to the manufacturer’s instructions. The glomerular apoptotic index was calculated as the number of glomeruli with at least one TUNEL-positive nucleus divided by the total number of glomeruli. All microscopic fields of each complete kidney section were quantified[25].

### Enzyme-linked immunosorbent assay (ELISA)

The levels of rat and human Angptl4 from serum and urine samples were determined via ELISA. For Angptl4 detection, the assay sample and buffer were incubated with an Angptl4-HRP conjugate in a pre-coated plate (Blue Gene Biotech, Shanghai, China) as previously described[12]. The color intensity was spectrophotometrically measured at 450 nm with an ELISA reader (Multiskan MK3, Thermo Labsystems, Vantaa, Finland).

### Immunostaining

Following collection, the rat and human kidney tissues were fixed in paraformaldehyde/lysine/periodate (PLP) solution for 2 hours, and the tissues were then incubated in 18% sucrose overnight, as previously described[26,27]. The tissues were then subsequently embedded in Tissue-Tek opti-mum cutting temperature compound (OCT) compound, snap-frozen in liquid nitrogen and cut with a freezing microtome (Thermo Cryotome E, Shandon, UK) to a thickness of 4 μm.

To determine the expression of Angptl4 in the glomeruli, its co-localization with podocytes, endothelium cells, mesangial cells, glomerular basement membrane (GBM) and injured podocytes in ADR rats was determined. Cryosections were stained with goat anti-rat/human Angptl4 (1:100, Santa Cruz Biotech, Santa Cruz, CA, USA), mouse anti-rat desmin (1:100, Abcam, New Territories, Hong Kong), mouse anti-rat synaptopodin (1:10, Progen, Heidelberg, Germany), mouse anti-rat laminin (1:400, Abcam, New Territories, Hong Kong), mouse anti-rat RECA-1 (1:10, Abcam, New Territories, Hong Kong) and mouse anti-rat OX-7 (1:200, Abcam, New Territories, Hong Kong) overnight at 4°C. The cryosections were then incubated with Alexa Fluor 488-conjugated donkey anti-goat IgG (1:200, Jackson ImmunoResearch, West Grove, PA, USA) and Alexa Fluor 594-conjugated donkey anti-mouse IgG (1:200, Jackson ImmunoResearch, West Grove, PA, USA). To exclude the non-specificity of this Angptl4 antibody for immunofluorescence, we analyzed negative controls using normal rats and secondary antibody only. The procedure and antibodies used for the human kidney tissue immunostaining were the same as the rat kidney tissue immunostaining.

Epifluorescence images were obtained with a Nikon microscope (Tokyo, Japan). All exposure settings were maintained constant for each group of kidneys. Images were sequentially captured via digital imaging of the entire sagittal section, including the cortex and outer medulla (10–15 images). The fluorescence intensity was measured by manually outlining the perimeters of 10 glomeruli per section and semi-quantifying the luminosity of the outlined regions with image analysis software (Image J, version 1.47, National Institutes of Health, Bethesda, MD, USA). A background correction was performed for each glomerulus by subtracting the average intensity of the non-stained regions (manually outlined) in the glomeruli. The co-localization ratio was analyzed with Image J software, using a co-localization plug-in to calculate the co-localization area, which was subsequently divided by the corresponding total area.

### Quantitative real-time PCR

RNA extraction from the glomeruli of rats, cDNA synthesis, and real-time PCR were performed using methods previously described[11]. The glomeruli of rats were isolated using the standard sieving method. The primer sequences for the real-time PCR were as follows: Angptl4, 5′-CCAATGGCCTTTCCCTGCCCTT-3′ and 5′- TTTTACGCTGCTGCCGTTGCC-3′. There were three replicates for each sample. The experimental cycle threshold (CT) values were normalized to glyceraldehyde-3-phosphate dehydrogenase (GAPDH) measured in the same plate, and the fold differences in gene expression were determined using the 2^-ΔΔCT^ method[28].

### Western blot analysis of urinary and glomerular Angptl4

The isolation and homogenates of the rat glomeruli and western blot analysis were performed as previously described[11]. The volume of centrifuged urine from the ADR rats and MCD patients was 20 μl, and the quantity of lysates from the ADR rats was 100 μg. For the detection of Angptl4, synaptopodin and desmin, the blots were incubated with goat anti-rat Angptl4 (Santa Cruz Biotech, Santa Cruz, CA, USA, 1:200), mouse anti-rat synaptopodin (1:50, Progen, Heidelberg, Germany) or mouse anti-rat desmin (1:100, Abcam, New Territories, Hong Kong). The following secondary antibodies were used: horseradish peroxidase-conjugated rabbit anti-goat IgG (Jackson ImmunoResearch, West Grove, PA, USA, 1:5000) or horseradish peroxidase-conjugated goat anti-mouse IgG (Jackson ImmunoResearch, West Grove, PA, USA, 1:5000). All western blot results were normalized to β-actin.

### Statistical analysis

All data are expressed as the means ± standard deviation (SD). Statistical analyses were conducted using one-way analysis of variance (ANOVA) with the least significant difference (LSD) t test, the two-sample t test and Spearman’s coefficient of correlation analysis using SPSS software (version 21.0; Chicago, IL, USA). A value of P<0.05 was considered significant, and P<0.01 was considered highly significant.

## Results

### Severe proteinuria and lipid metabolism disorders were ameliorated by tacrolimus in ADR rats

**Fig 1:**
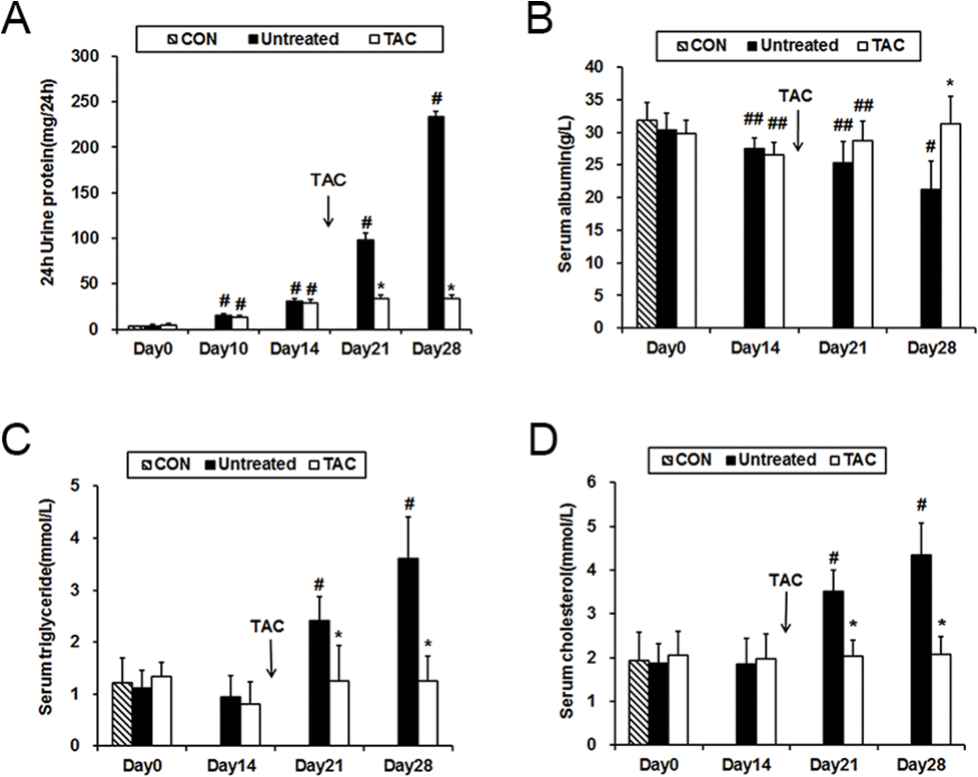
Tacrolimus-treated rats exhibited ameliorated ADR-induced proteinuria and lipid metabolism disorders. **(A)** Twenty-four hour urinary protein excretion in ADR rats. **(B)** Serum albumin levels in ADR rats. **(C)** Serum triglyceride levels in ADR rats. **(D)** Serum cholesterol levels in ADR rats. Con, normal rats; Untreated, ADR rats without treatment; TAC, ADR rats with tacrolimus treatment. ##P<0.05 compared with normal rats; #P<0.01 compared with normal rats; *P<0.01 compared with untreated ADR rats. The arrow indicates that tacrolimus treatment was initiated on day 14.

In this study, heavy proteinuria, hypoalbuminemia and hyperlipidemia were the main clinical features of nephrotic syndrome identified in ADR rats (Fig 1). Furthermore, no evidence indicating that the pathological diagnosis was FSGS during the time course of this experiment was uncovered, which indicated that a successful MCD experimental model was established similar to previous research[23]. Tacrolimus, an immunosuppressant used to treat many types of proteinuric kidney diseases[29,30], was administered on day 14 when proteinuria occurred in the ADR rats. After tacrolimus treatment, 24-hour proteinuria excretion was significantly decreased on days 21 and 28 compared with the untreated group (Fig 1A). In accordance with the markedly reduced proteinuria, the serum albumin levels increased (Fig 1B), and the serum triglycerides (Fig 1C) and serum cholesterol decreased (Fig 1D) after tacrolimus administration.

### Increased podocyte injury and apoptosis induced by ADR were ameliorated by tacrolimus administration

**Fig 2:**
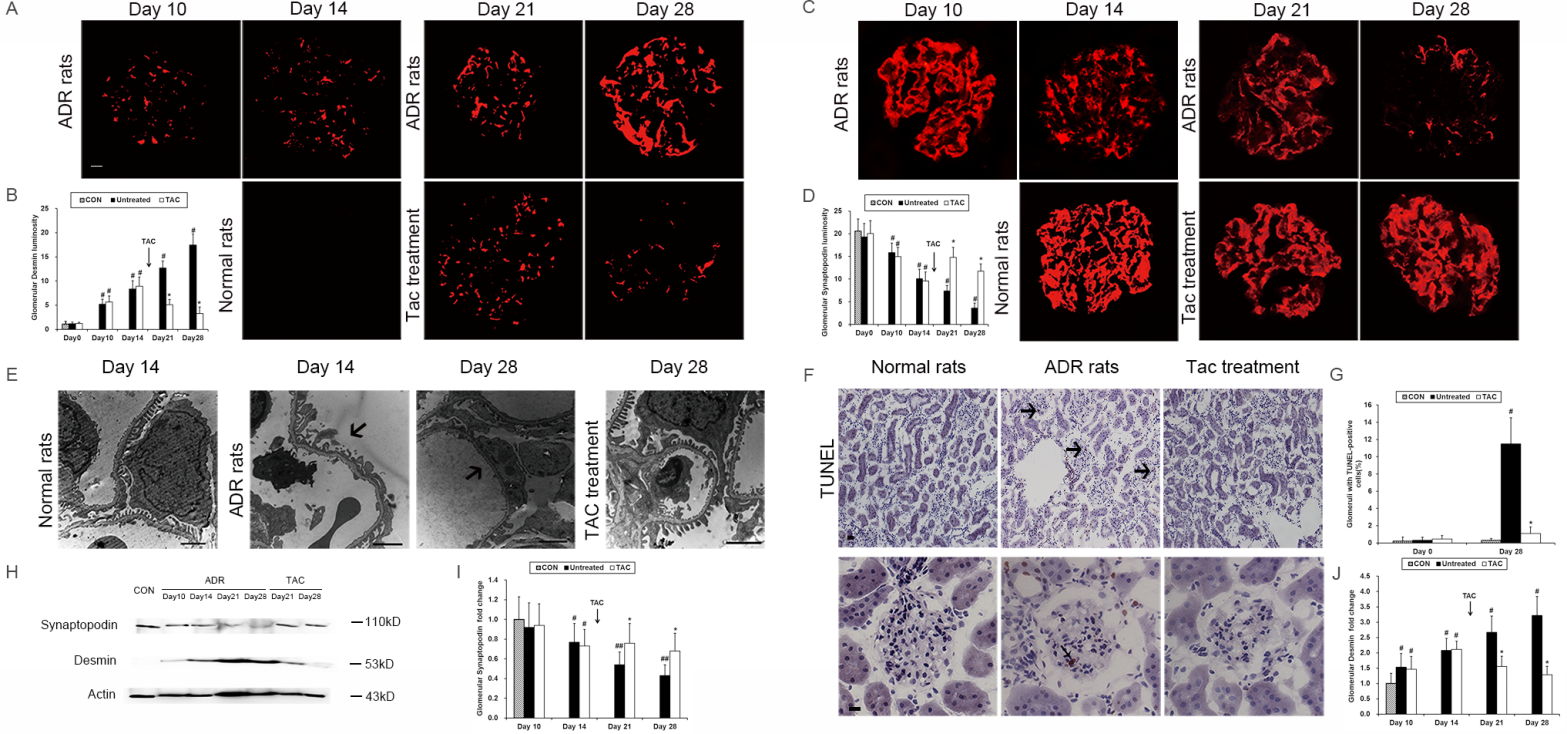
Tacrolimus promoted podocyte repair in ADR rats. **(A)** Immunofluorescence of glomerular desmin in normal, tacrolimus-treated and untreated ADR rats. Scale bars = 50 μm. **(B)** Quantification of the fluorescence staining intensities of glomerular desmin in normal, tacrolimus-treated and untreated ADR rats. **(C)** Immunofluorescence of glomerular synaptopodin in normal, tacrolimus-treated and untreated ADR rats. **(D)** Quantification of the fluorescence staining intensities of glomerular synaptopodin in normal, tacrolimus-treated and untreated ADR rats. **(E)** Transmission electron microscopy of normal, tacrolimus-treated and untreated ADR rats. Foot process effacements are indicated by the black arrows. Scale bars = 2 μm. **(F)** TUNEL assay of glomeruli from ADR rats. A TUNEL-positive cell is indicated by the black arrow. Scale bars = 50 μm. **(G)** Quantification of the TUNEL assay of the glomeruli from ADR rats. **(H)** Western blot of glomerular synaptopodin and desmin expression in ADR rats. **(I)** Quantification of the western blot of glomerular synaptopodin expression in ADR rats. **(J)** Quantification of the western blot of glomerular desmin expression in ADR rats. Con, normal rats; Untreated, ADR rats without treatment; TAC, ADR rats with tacrolimus treatment. ##P<0.05 compared with normal rats; #P<0.01, compared with normal rats; *P<0.01 compared with untreated ADR rats.

We subsequently investigated podocyte injury by evaluating the expression of desmin, a biomarker of injured podocytes, and synaptopodin, a biomarker for normal podocytes. Our data indicated that desmin expression was significantly upregulated (Fig 2A and 2B), whereas synaptopodin expression was clearly downregulated in the ADR rats compared with the normal rats (Fig 2C and 2D). Tacrolimus administration dramatically prevented the enhanced expression of desmin (Fig 2A and 2B) and reversed the reduction of synaptopodin expression (Fig 2C and 2D) induced by ADR compared with untreated group on days 21 and 28. Similar results were obtained for the ADR rats via western blot analysis (Fig 2H–2J). In addition, the same evidence was also demonstrated via electron microscopy as shown in Fig 2E. Extensive podocyte foot process effacement induced by ADR was markedly ameliorated after tacrolimus treatment (Fig 2E). Moreover, the increased glomerular apoptotic cells were significantly reduced after tacrolimus treatment (Fig 2F and 2G). These data demonstrated that tacrolimus promoted podocyte repair and prevented apoptosis following injury induced by ADR.

### Increased Angptl4 expression occurred earlier and reached a peak more rapidly than desmin and proteinuria in ADR rats, and tacrolimus diminished glomerular and urinary Angptl4 expression

**Fig 3:**
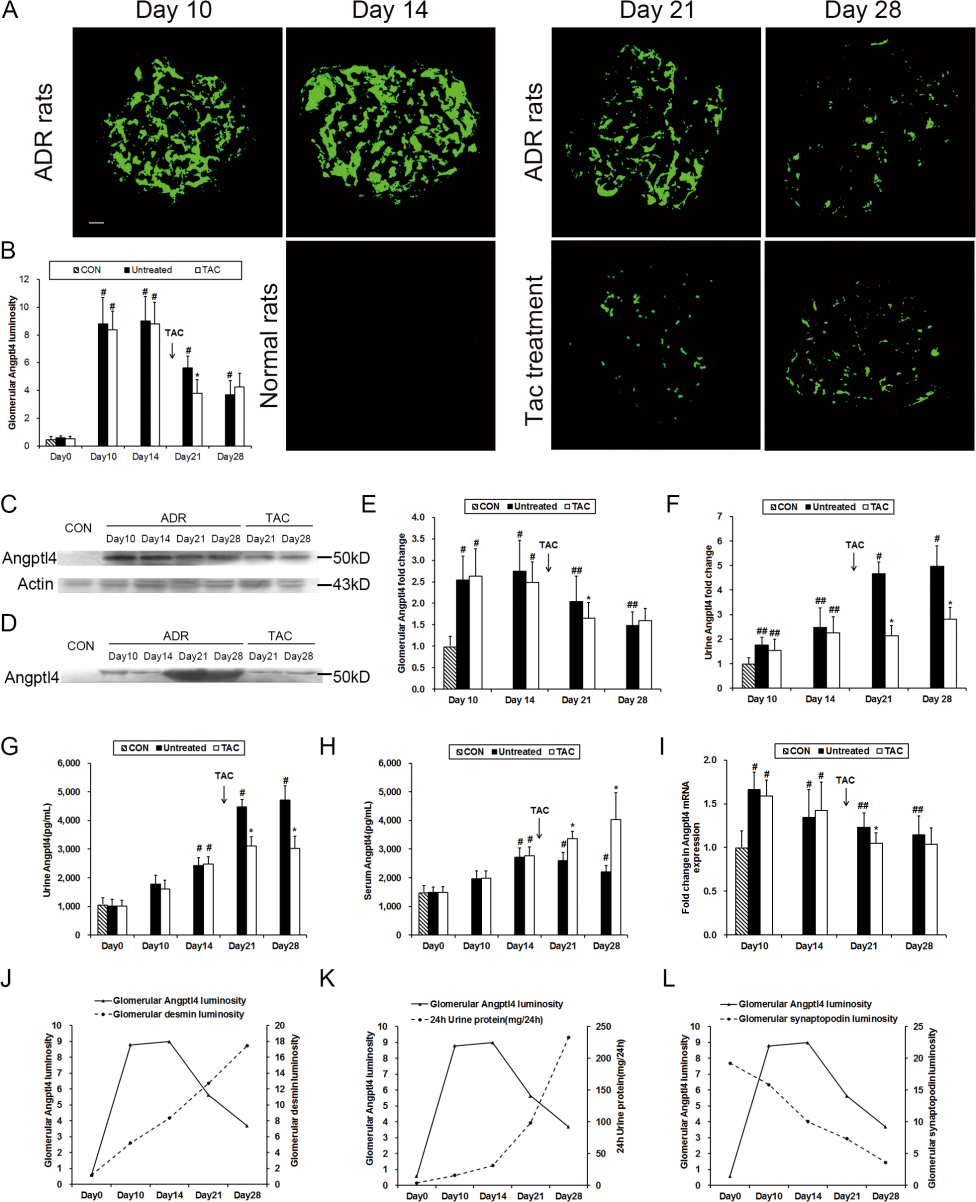
Angptl4 was expressed at an earlier stage of podocyte injury in ADR rats, and tacrolimus diminished glomerular and urinary Angptl4 expression. **(A)** Immunofluorescence of glomerular Angptl4 in normal, tacrolimus-treated and untreated ADR rats. Scale bars: 50 μm. **(B)** Quantifications of the fluorescence staining intensities of glomerular Angptl4 in normal, tacrolimus-treated and untreated ADR rats. **(C)** Western blot of glomerular Angptl4 expression in ADR rats. **(D)** Western blot of urine Angptl4 excretion in ADR rats. **(E)** Quantification of the western blot of glomerular Angptl4 expression in ADR rats. **(F)** Quantification of the western blot of urine Angptl4 excretion. **(G)** Urine Angptl4 ELISA for ADR rats. **(H)** Serum Angptl4 ELISA for ADR rats. **(I)** Quantification of the real-time PCR of glomerular Angptl4 in ADR rats. **(J)** Relationship between glomerular Angptl4 and glomerular desmin in ADR rats. **(K)** Relationship between glomerular Angptl4 and 24-hour urinary protein excretion in ADR rats. **(L)** Relationship between glomerular Angptl4 and glomerular synaptopodin in ADR rats. Con, normal rats; Untreated, ADR rats without treatment; TAC, ADR rats with tacrolimus treatment. ##P<0.05 compared with normal rats; #P<0.01 compared with normal rats; *P<0.01 compared with untreated ADR rats. The arrow indicates that tacrolimus treatment was initiated on day 14.

There was scant Angptl4 expression within the glomeruli in normal rats (Fig 3A). Immunofluorescence indicated that the glomerular Angptl4 expression was upregulated in the ADR rats. The glomerular Angptl4 intensity reached its peak on day 14 and gradually decreased on days 21 and 28. Tacrolimus notably diminished the glomerular Angptl4 expression on day 21; however, it exhibited slightly increased expression on day 28 (Fig 3A and 3B). Moreover, similar trends were indicated via western blot analysis (Fig 3C and 3E) and quantitative real-time PCR (Fig 3I). Additionally, increased Angptl4 was excreted into the urine in the ADR rats, and tacrolimus administration decreased this excretion (Fig 3D and 3F). A similar trend in urinary Angptl4 expression was also confirmed via ELISA (Fig 3G). However, tacrolimus treatment increased the circulating Angptl4 expression in the ADR rats on days 21 and 28 (Fig 3H).

To determine the relationships between glomerular Angptl4 expression and podocyte injury or proteinuria, we created line charts as shown in Fig 3J–3L. The Angptl4 expression was rapidly upregulated to its peak prior to desmin expression and proteinuria (Fig 3J and 3K), and the increase in the glomerular Angptl4 expression was more significant than the decrease in the synaptopodin expression during an earlier stage of podocyte injury (Fig 3L). These findings indicated that increased Angptl4 expression may represent an earlier injury biomarker than desmin and proteinuria in ADR rats.

### Glomerular Angptl4 was mainly located in injured podocytes in ADR rats

**Fig 4:**
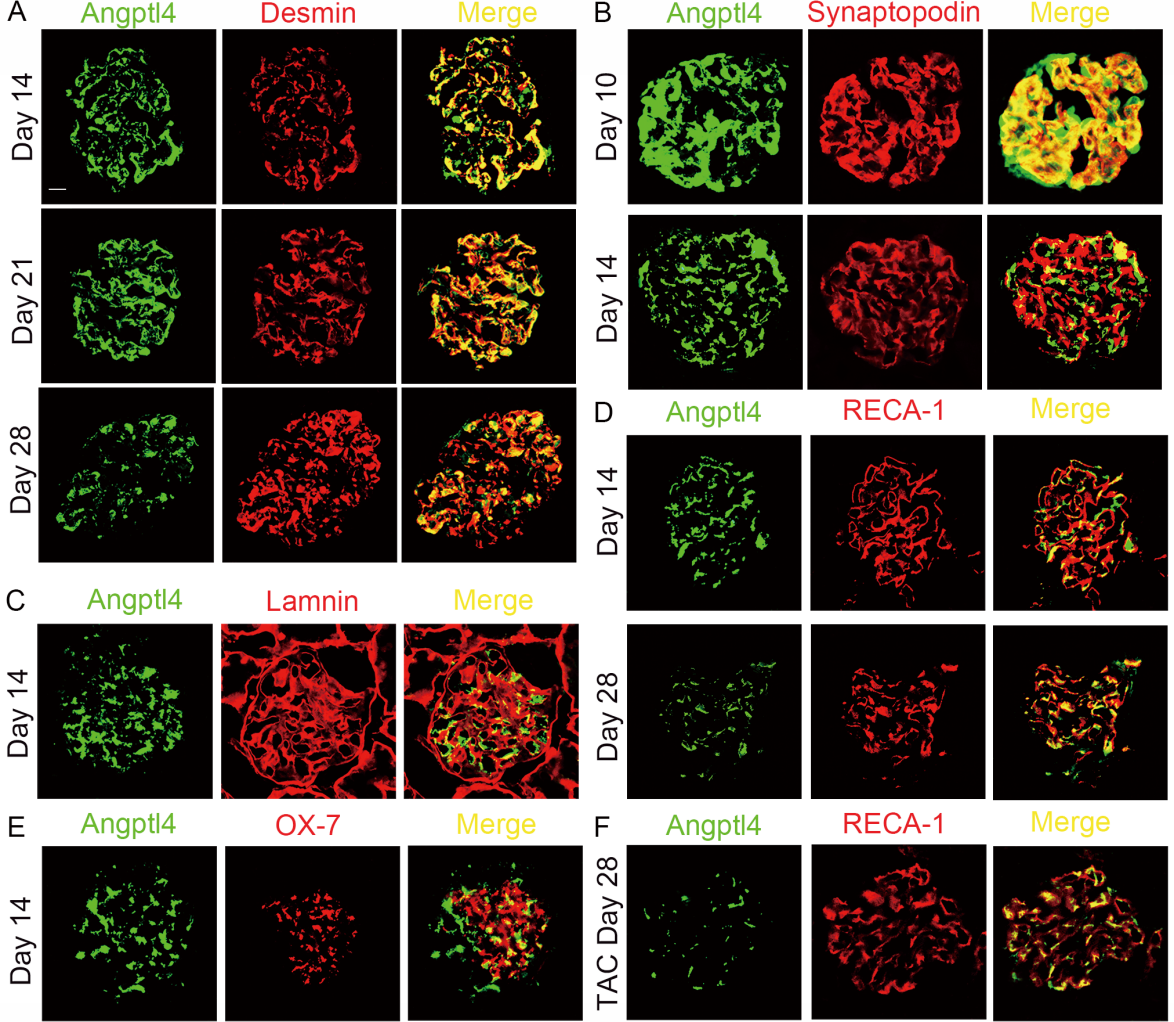
The majority of glomerular Angptl4 was secreted by injured podocytes in ADR rats. **(A)** Immunofluorescence of glomerular Angptl4 and desmin, an injured podocyte marker, in ADR rats on days 14, 21 and 28. **(B)** Immunofluorescence of glomerular Angptl4 and synaptopodin, a normal podocyte marker, in ADR rats on days 10 and 14. **(C)** Immunofluorescence of glomerular Angptl4 and laminin, a GBM marker, in ADR rats on day 14. **(D)** Immunofluorescence of glomerular Angptl4 and RECA-1, an endothelial cell marker, in ADR rats on days 14 and 28. **(E)** Immunofluorescence of glomerular Angptl4 and OX-7, a mesangial cell marker, in ADR rats on day 14. **(F)** Immunofluorescence of glomerular Angptl4 and RECA-1 in ADR rats with tacrolimus treatment on day 28. Scale bars: 50 μm. TAC, ADR rats with tacrolimus treatment.

To determine the type of glomerular cell that secreted Angptl4 in ADR rats, we co-stained glomerular Angptl4 with the following markers: RECA-1, an endothelial marker; OX-7, a mesangial cell marker; laminin, a GBM marker; synaptopodin, a normal podocyte marker; and desmin, an injured podocyte marker. Angptl4 effectively co-localized with the normal podocyte marker on day 10 (57.62% of Angptl4 was coincident with synaptopodin, Fig 4B) and was not co-localized with it on day 14 (18.77% of Angptl4 was co-localized with synaptopodin, Fig 4B). However, Angptl4 persistently co-localized with the injured podocyte marker on day 14 (63.52% of Angptl4 was co-localized with desmin, Fig 4A) and day 21 (74.26% of Angptl4 was co-localized with desmin, Fig 4A). Glomerular Angptl4 gradually separated from the injured podocyte marker, desmin, on day 28 (66.73% of Angptl4 was co-localized with desmin, Fig 4A). Furthermore, Angptl4 co-staining with laminin indicated that glomerular Angptl4 was rarely localized with this GBM marker in the ADR rats on day 14 (22.87% Angptl4 co-localized with laminin, Fig 4C). The glomerular Angptl4 expression in the ADR rats was separate from the endothelium (Fig 4D) and mesangial cells (Fig 4E) on day 14. Nevertheless, the co-localization of glomerular Angptl4 and RECA-1 was 21.32% (Fig 4D) in the untreated ADR rats on day 28; however, the co-localization was 32.45% (Fig 4F) in the tacrolimus-treated ADR rats with increasing serum Angptl4 expression(Fig 3H). This finding may be explained by the increased glomerular Angptl4 expression that may have originated from circulating Angptl4 that co-localizes with endothelial cells, which is consistent with previous research[31].

### Increased expression of glomerular and urinary Angptl4 was also confirmed in human MCD patients and related to podocyte injury and 24-hour urine protein levels

**Fig 5:**
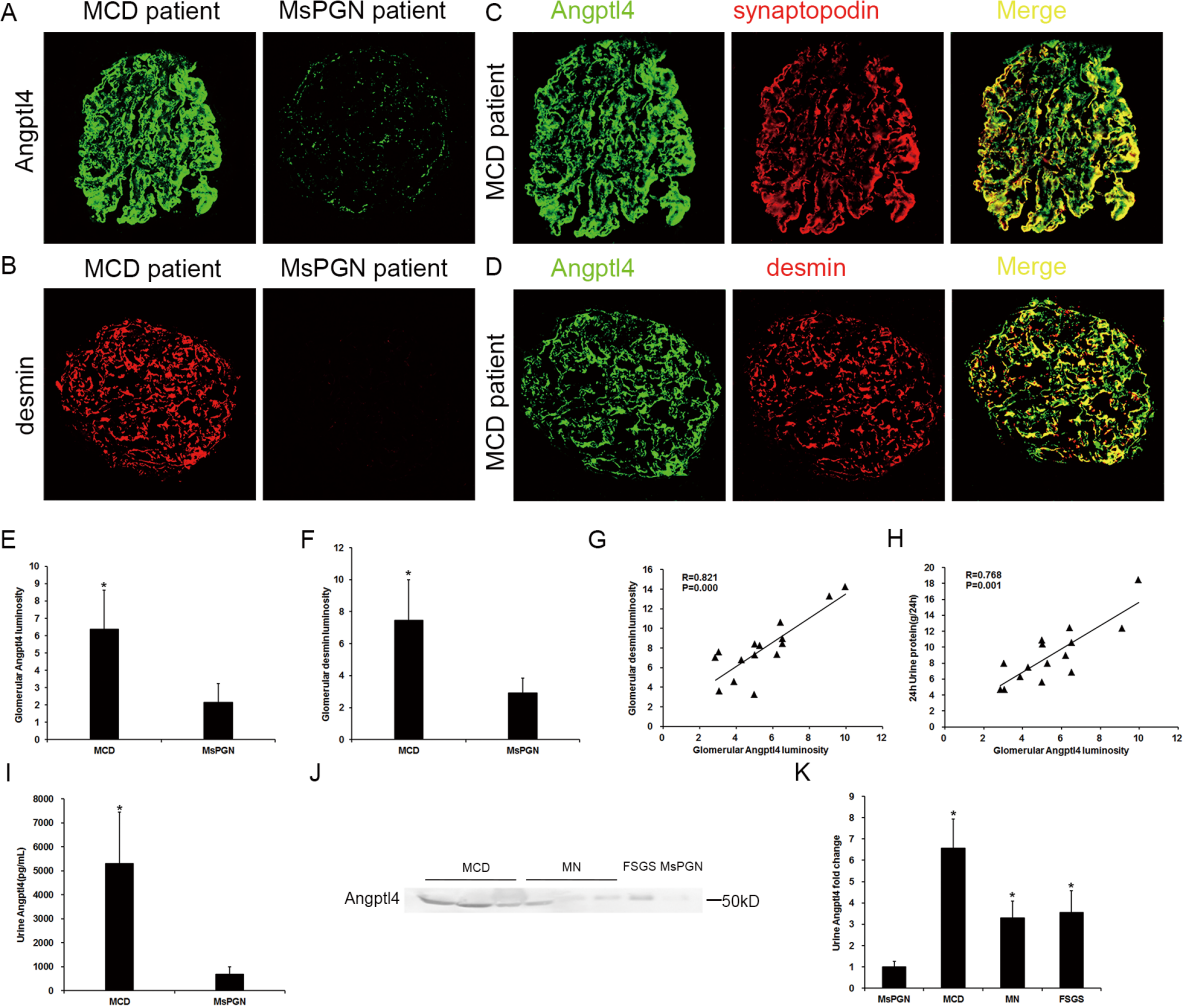
Increase in Angptl4 expression in human MCD patients and associations with desmin, synaptopodin and proteinuria. **(A and B)** Immunofluorescence of glomerular Angptl4 and desmin in MCD and MsPGN patients with similar nephrotic-range proteinuria (magnification, 200X). **(C)** Immunofluorescence of glomerular Angptl4 and synaptopodin in MCD patients (magnification, 200X). **(D)** Immunofluorescence of glomerular Angptl4 and desmin in MCD patients (magnification, 200X). **(E and F)** Quantifications of the fluorescence staining intensities of glomerular Angptl4 and desmin in MCD and MsPGN patients. **(G)** Scatter diagram of glomerular Angptl4 and desmin in MCD patients. **(H)** Scatter diagram of Angptl4 and 24-hour urinary protein in MCD patients. **(I)** Urine Angptl4 ELISA for MCD and MsPGN patients with similar nephrotic-range proteinuria. **(J)** Western blot of urinary Angptl4 excretion in MCD, MN, FSGS and MsPGN patients (MCD 1,2,3, MN 1,2,3, FSGS 1 and MsPGN 4 in Table 1 are shown in the image) with similar nephrotic-range proteinuria. **(K)** Quantification of the western blot of urinary Angptl4 excretion in MCD, MN, FSGS and MsPGN patients. *P<0.01 compared with MsPGN patients.

To further confirm glomerular Angptl4 expression in MCD, we stained for Angptl4, desmin and synaptopodin in samples obtained from nephrotic syndrome patients with MCD (n = 15), MN (n = 5), FSGS (n = 5) and mesangial proliferative glomerulonephritis (MsPGN, n = 5). The baseline characteristics of these patients are described in Table 1. A comparison of 5 patients with similar nephrotic-range proteinuria (5 with MCD and 5 with MsPGN) indicated that the Angptl4 and desmin expression levels were markedly upregulated in the MCD patients compared with the MsPGN patients (Fig 5A, 5B, 5E and 5F). These findings confirmed that enhanced Angptl4 expression was associated with podocyte injury. In the patients with MCD (n = 15), glomerular Angptl4 was effectively co-localized with the normal and injured podocyte markers (Fig 5C and 5D), which was consistent with the experimental MCD model.

**Table 1:** Baseline characteristics of the enrolled patients.

Patient	Age (years)	Gender	24h Urine protein (g/24 h)	Serum triglyceride (mmol/L)	Serum cholesterol (mmol/L)
MCD1	29	M	4.64	2.41	5.84
MCD2	44	F	7.95	2.82	11.73
MCD3	60	F	4.65	1.35	5.45
MCD4	24	M	36.20	2.45	8.85
MCD5	62	M	2.80	1.82	9.37
MCD6	49	F	12.42	2.01	8.99
MCD7	26	M	7.45	1.87	15.11
MCD8	68	M	6.81	1.72	7.34
MCD9	46	M	10.92	3.32	8.18
MCD10	30	F	5.97	3.40	8.84
MCD11	60	F	6.25	2.39	7.63
MCD12	68	M	6.86	0.98	7.40
MCD13	27	F	7.70	1.76	8.07
MCD14	19	M	17.63	3.22	11.08
MCD15	21	F	15.76	3.47	14.36
MN1	31	F	3.74	1.64	5.96
MN2	36	F	8.30	4.05	10.39
MN3	58	F	6.40	2.84	9.98
MN4	31	M	33.66	2.82	8.69
MN5	36	M	18.98	1.84	8.34
FSGS1	48	F	8.58	3.35	15.67
FSGS2	23	M	22.80	2.94	10.07
FSGS3	56	M	19.63	4.51	18.51
FSGS4	26	M	11.07	4.31	14.58
FSGS5	64	M	10.64	2.18	7.44
MsPGN1	43	F	1.31	0.72	3.86
MsPGN2	38	F	0.18	1.02	3.53
MsPGN3	31	M	2.34	1.08	5.21
MsPGN4	60	M	3.36	1.38	5.73
MsPGN5	48	F	17.91	4.34	15.55

M, male; F, female.

A correlative analysis indicated positive correlations between glomerular Angptl4 and desmin expression (R = 0.821, P = 0.000, Fig 5G) as well as between glomerular Angptl4 expression and proteinuria (R = 0.811, P = 0.000, Fig 5H) in the 15 MCD patients, which indicated that glomerular Angptl4 expression may represent a potential biomarker for evaluating podocyte injury.

Further investigation of urinary Angptl4 excretion via ELISA indicated that urinary Angptl4 was significantly upregulated in the MCD patients as compared to the MsPGN patients (Fig 5I). Similar results were noted in the western blot analysis between the patients with podocytopathy (MCD, MN and FSGS) and the patients with MsPGN (Fig 5J and 5K). These findings indicated that highly increased Angptl4 expression may be prone to occur in patients with podocytopathy.

## Discussion

Podocyte injury plays central roles in proteinuria and kidney dysfunction, therefore, identifying specific biomarker to evaluate earlier podocyte injury is highly desirable. Angptl4, which can be detected in urine and is related to proteinuria, facilitated the prediction of podocyte injury and the evaluation of treatment efficacy in patients with podocytopathy[10,11,31]. In this study, we demonstrated that glomerular and urinary Angptl4 were significantly increased during the earlier stage of podocyte injury and were most likely associated with podocyte injury and heavy proteinuria in both ADR rats and MCD patients. Furthermore, the upregulation of glomerular Angptl4 expression was primarily the result of injured podocyte secretion. As the podocyte injury worsened, the glomerular Angptl4 expression reached its peak level and subsequently decreased; however, its expression level remained higher in the ADR rats compared to the control rats. The Angptl4 upregulation occurred only when podocyte was mainly damaged since we didn’t observe little Angptl4 upregulation in MsPGN patients. Our study demonstrated that tacrolimus treatment markedly reduced glomerular and urinary Angptl4 expression during an earlier stage, which was accompanied by a sustained increase in serum Angptl4, a reduction in proteinuria and the promotion of podocyte repair. Therefore, this study represents the first evidence that Angptl4 may serve as a specific biomarker for the evaluation of earlier podocyte injury not only in a MCD rat model, but also in MCD patients. And tacrolimus may target Angptl4 to reduce proteinuria and promote podocyte repair in MCD.

Angptl4 is expressed in certain tissues, such as adipose and liver tissues, and it is upregulated after fasting and hypoxia[9,32]. In addition, glomerular Angptl4 expression was low in normal kidneys in the present study. Recent research has demonstrated that podocyte-secreted Angptl4 induces proteinuria and circulating Angptl4 mediates hypertriglyceridemia[10,31]. In addition, a previous study has reported that nearly all Angptl4 expression is co-localized with podocytes on day 6 in a puromycin aminonucleoside (PAN) rat model[10]. However, only a 74% overlap was identified between Angptl4 staining and injured podocytes in the ADR rats in the current study. One potential explanation for this difference may be the different animal models, antibodies and time points examined. In addition, it is possible that Angptl4 expression was higher near the GBM rather than within the injured podocytes. Furthermore, double immunofluorescence demonstrated that injured podocytes were the primary sources of the additional Angptl4 (which exhibited an approximate 70% overlap with these cells, and this level remained stable). Furthermore, normal podocytes were not the major cell type that secreted Angptl4 (which exhibited an approximate 58% overlap with these cells that decreased to 19% over time). This study was the first to demonstrate that glomerular Angptl4 may primarily be secreted by injured podocytes in ADR nephropathy.

In the present study, urinary Angptl4 excretion exhibited similar trends to glomerular Angptl4 expression. Glomerular Angptl4 expression correlated with proteinuria during the early stage, as proteinuria continued to increase and glomerular Angptl4 levels declined on day 28. A possible reason for this finding may be that the ADR rat model involves multiple pathways in which Angptl4 is one of the key components, and proteinuria in an ADR rat model is multifactorial and involves other key components during the later stage. The decrease in urinary Angptl4 excretion and the increase in serum Angptl4 excretion following the amelioration of podocyte injury may be explained by two primary factors. First, podocyte-secreted Angptl4, which has been demonstrated to induce proteinuria in podocyte-specific Angptl4 transgenic mice and rats, migrated downwards as podocyte repair improved[10]. Second, recent research has indicated that circulating Angptl4 secreted by the liver, adipose tissue and other peripheral tissues binds to glomerular epithelial cells and reduces proteinuria[31]. However, it remains unclear if additional Angptl4 is excreted into the urine following the inhibition of podocyte injury. As previously discussed, glomerular Angptl4 was upregulated significantly earlier than the changes in desmin and synaptopodin in ADR rats, which suggests that podocyte injury was induced by Angptl4 and that the over-expression of glomerular Angptl4 may result in foot process effacement and cytoskeleton damage. This study provides the first evidence to suggest that urinary Angptl4 may represent a potential biomarker for the prediction of podocyte injury in MCD. In addition, positive correlations were identified between urinary Angptl4 and podocyte injury in human MCD patients. However, because our results were limited by the number of MCD patients included, further investigation must be undertaken in the future.

Emerging evidence has indicated that tacrolimus acts on calcineurin, a central signaling controller in eukaryotes[17], which results in multi-systemic side effects such as hypertension and pathoglycemia[18,19]. Therefore, investigation of the downstream targets of tacrolimus action on refractory MCD could identify new treatment options for patients with this disease. Moreover, the immunosuppressive effects of tacrolimus[33,34] could explain, in part, its therapeutic effects in autoimmune diseases and transplantation[35,36]. In addition, calcineurin inhibitors could also reduce proteinuria in Alport’s syndrome, which is a non-immunological disease[37]. Therefore, we hypothesized that tacrolimus may have the additional benefit of anti-proteinuric activity in MCD independent of its immunological effects and that it may directly affect podocytes. Positive correlations between glomerular Angptl4 and podocyte injury as well as proteinuria were identified in MCD patients. Surprisingly, Angptl4 expression reached its peak substantially earlier than desmin and urine protein excretion in ADR rats. Furthermore, these findings were ameliorated by tacrolimus treatment, which indicated that tacrolimus reduced proteinuria and podocyte injury through its effects on Angptl4. Tacrolimus significantly reduced glomerular Angptl4 expression and urinary Angptl4 excretion; however, it increased serum Angptl4 expression and alleviated defects in lipid metabolism following continuous treatment in ADR rats. These findings may have been the result of the binding of circulating Angptl4 to glomerular endothelial cells, which may have reduced proteinuria through Angptl4 negative feedback loops[31]. Thus, we speculated that tacrolimus reduced podocyte-secreted Angptl4 and increased circulating Angptl4 to ameliorate proteinuria and podocyte injury. In addition, we observed increased urinary excretion of Angptl4 at days 21 and 28 in the ADR rats. We suspected that some of the urinary Angptl4 might have come from the circulation as Angptl4 expression declined in the glomeruli on days 21 and 28.

Previous work[31] has demonstrated that circulating Angptl4 induced hypertriglyceridemia in PHN and PAN rat models, and our previous study reported that tacrolimus reduces serum triglycerides in a PHN rat model[11]. In the current study, we determined that tacrolimus improved defects in lipid metabolism, and we presumed that these findings were the result of tacrolimus-induced activation of a specific pathway that could inhibit hypertriglyceridemia and hypercholesterolemia via circulating Angptl4. However, further investigation is needed to validate this hypothesis.

The upstream mechanisms that stimulate increased podocyte Angptl4 expression are unknown and require further investigation. In particular, future studies should assess if the reduction in glomerular and urine Angptl4 expression and podocyte injury are closely related in other experimental models with massive proteinuria. Because Angptl4 could was detected in the urine of MCD patients and urine Angptl4 was related to podocyte injury in these individuals, further studies are required to determine if urine Angptl4 represents a non-invasive maker for podocyte injury identification in other podocytopathy cases.

In conclusion, this study provided the first evidence that urine Angptl4 predicted podocyte injury in both experimental and human MCD diseases. Moreover, this study showed that tacrolimus decreased podocyte-secreted Angptl4 to reduce proteinuria in an MCD rat model. However, these findings cannot definitively demonstrate the relationship between Angptl4 and podocyte injury, and our conclusions cannot exclude the direct effects of tacrolimus on podocytes. Thus, additional research is required to clarify these remaining issues.

## Supporting Information

S1 Fig Negative controls and normal control in ADR rats. **(A)** Negative control for ADR rats (magnification, 200X). ADR, kidney tissue from ADR rats on day 10 stained with goat anti-rat Angptl4 antibody and the subsequent secondary antibody; IgG negative control, kidney tissue from ADR rats on day 10 stained with goat IgG and the subsequent secondary antibody; PBS negative control, kidney tissue from ADR rats on day 10 stained with PBS and the subsequent secondary antibody; Normal control, kidney tissue from normal rats stained with goat anti-rat Angptl4 antibody and the subsequent secondary antibody.
(TIF)
