# Supplementary figures and images for: Angiopoietin-Like-4, a Potential Target of Tacrolimus, Predicts Earlier Podocyte Injury in Minimal Change Disease
Source: PLoS One. 2015 Sep 9;10(9):e0137049. doi: 10.1371/journal.pone.0137049 (PMC4564140; doi:10.1371/journal.pone.0137049)

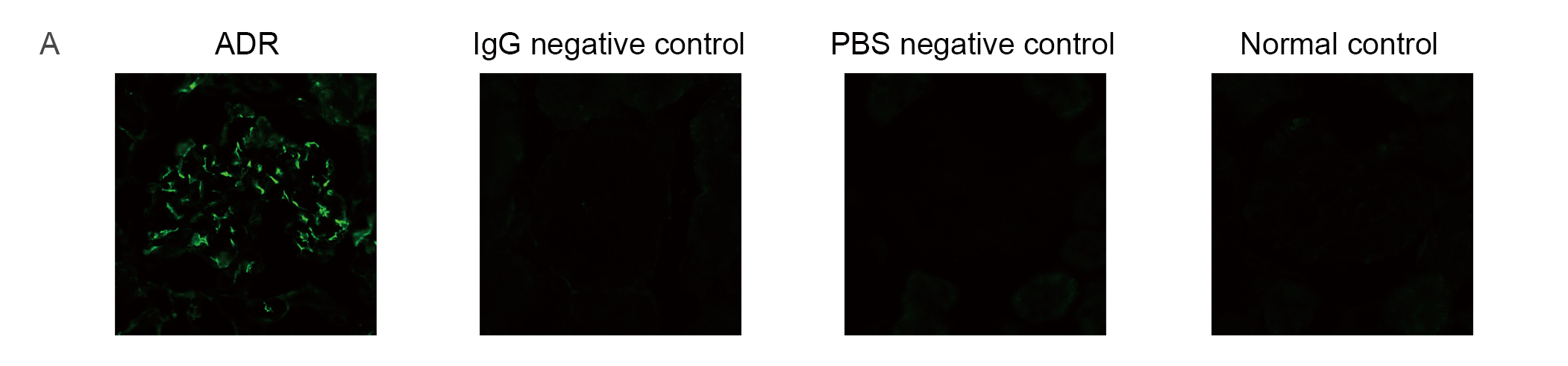

Supplement: S1 Fig — (A) Negative control for ADR rats (magnification, 200X). ADR, kidney tissue from ADR rats on day 10 stained with goat anti-rat Angptl4 antibody and the subsequent secondary antibody; IgG negative control, kidney tissue from ADR rats on day 10 stained with goat IgG and the subsequent secondary antibody; PBS negative control, kidney tissue from ADR rats on day 10 stained with PBS and the subsequent secondary antibody; Normal control, kidney tissue from normal rats stained with goat anti-rat Angptl4 antibody and the subsequent secondary antibody. (TIF) [file pone.0137049.s001.tif]
